# Targeting BCMA to Treat Multiple Myeloma: Updates From the 2021 ASH Annual Meeting

**DOI:** 10.3389/fimmu.2022.839097

**Published:** 2022-03-07

**Authors:** Ruiting Guo, Wenyi Lu, Yi Zhang, Xinping Cao, Xin Jin, Mingfeng Zhao

**Affiliations:** ^1^ First Center Clinic College of Tianjin Medical University, Tianjin, China; ^2^ Department of Hematology, Tianjin First Central Hospital, Tianjin, China

**Keywords:** B-cell maturation antigen, CAR-T cell therapy, antibody-drug conjugates, bispecific T-cell engagers, immunotherapy, multiple myeloma

## Abstract

With the gradual improvement of treatment regimens, the survival time of multiple myeloma (MM) patients has been significantly prolonged. Even so, MM is still a nightmare with an inferior prognosis. B-cell maturation antigen (BCMA) is highly expressed on the surface of malignant myeloma cells. For the past few years, significant progress has been made in various BCMA-targeted immunotherapies for treating patients with RRMM, including anti-BCMA mAbs, antibody-drug conjugates, bispecific T-cell engagers, and BCMA-targeted adoptive cell therapy like chimeric antigen receptor (CAR)-T cell. The 63rd annual meeting of the American Society of Hematology updated some information about the application of BCMA in MM. This review summarizes part of the related points presented at this conference.

## 1 Introduction

**Figure 1:**
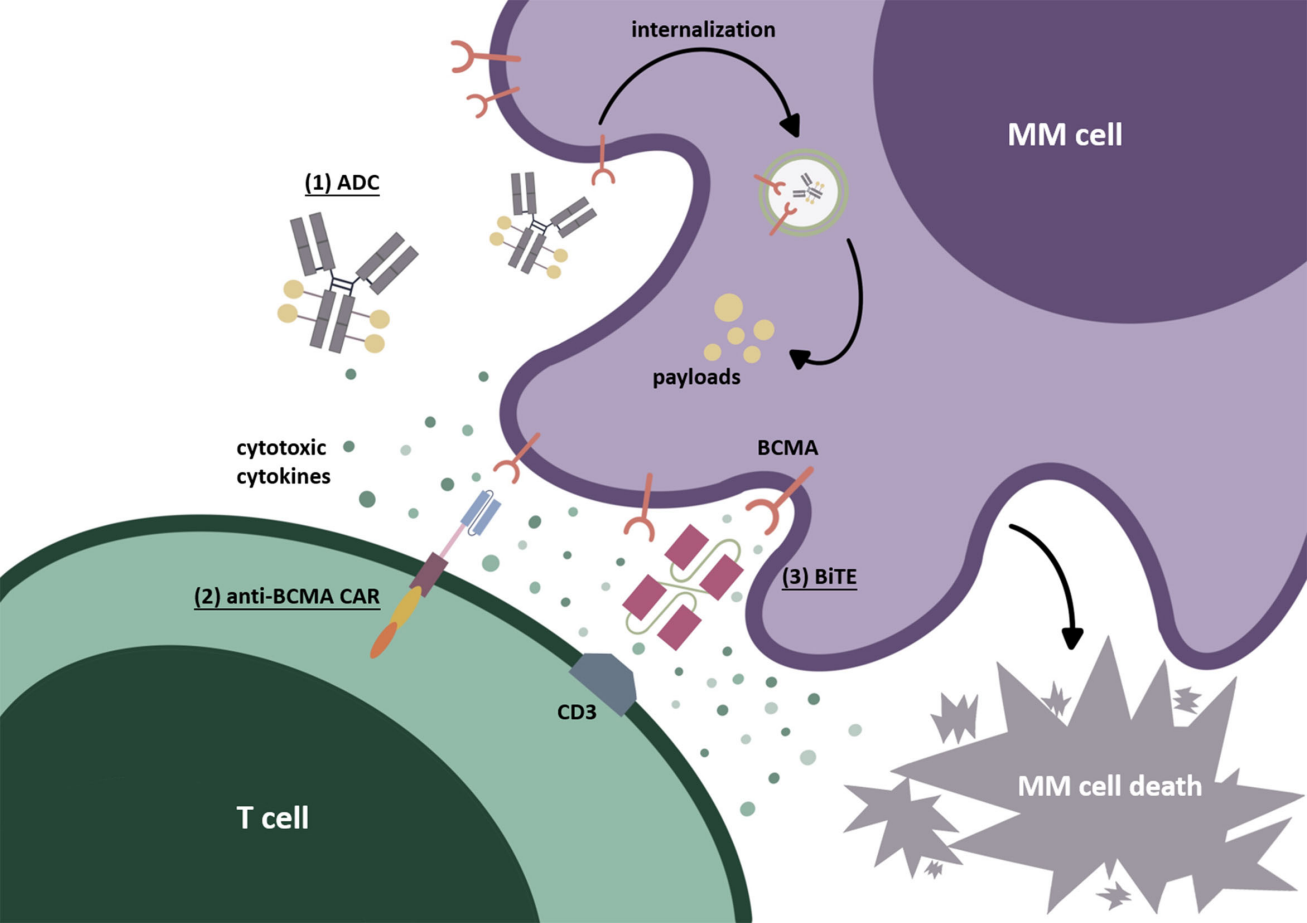
BCMA-targeted immunotherapies. (1) Antibody-drug conjugate (ADC). After identifying BCMA on the cell surface, ADC internalizes into myeloma cells. Through the degradation by lysosomes or endosomes, the payloads are released, resulting in cytotoxicity. (2) Chimeric antigen receptor (CAR) T cell. The second-generation CAR commonly used today is mainly composed of an extracellular recognition domain (the most commonly used is scFv), a spacer, a transmembrane part, and intracellular structures (costimulatory domain such as CD28 or 4-1BB and an activating domain CD3-zeta). The recognition domain binds to BCMA on the myeloma cell surface as signal 1. The costimulatory domain (CD28 or 4-1BB) is then “aroused” to send signal 2, which is beneficial to CAR-T-cell activation and to prevent their disability. Finally, signals 1 and 2 are transmitted to the CD3-zeta domain to induce CAR-T-cells’ final activation. (3) Bispecific T-cell engager (BiTE). BiTEs can target BCMA on MM tumor cells and CD3ε domain of TCR on T cells simultaneously. After causing the binding of T cells to myeloma cells, the cytotoxic T cells can be activated and secrete cytotoxic factors, thus producing the cytolethal effect.

In recent years, the strategies for treating multiple myeloma (MM) have advanced across the board (1). In the second half of the previous century, Melphalan chemotherapy combined with steroids use such as prednisone or dexamethasone was the basic therapeutic regimen for treating MM (2). Later, with the widespread application of proteasome inhibitor (PI) and immunomodulatory drug (IMiD), the prognosis of MM patients has been dramatically improved. From the finding of targeted monoclonal antibodies (mAbs), which have a favorable curative effect in MM (3, 4), the treatment for MM has shifted to focus on multiple immunotherapies, and their most salient point was undoubtedly targeted immunotherapy. B-cell maturation antigen (BCMA/CD269), which belongs to TNF receptor superfamily member 17 (5), is highly selectively expressed on the surface of MM cells, as the ideal target of majority targeted agents studied currently for the patients with MM (6), such as anti-BCMA mAbs, antibody-drug conjugates (ADCs), bispecific T-cell engagers (BiTEs), and BCMA-targeted adoptive cell therapy like chimeric antigen receptor (CAR)-T cell (**Figure 1**). The data relating to the efficacy and safety of these targeted immunotherapy products have gotten more comprehensive based on a great number of preclinical and clinical trials. The 63rd annual conference of the American Society of Hematology (ASH) showed us the latest progress of multiple anti-BCMA immunotherapies. This review aims to summarize some of the main points in this meeting about the application of BCMA in MM, with a special focus on clinical achievements.

## 2 Progress of the Mechanism Related to BCMA

Under physiological conditions, BCMA is mainly expressed on plasmablasts (7) and terminally differentiated plasma cells (PCs) (8). In the pathological case, BCMA is expressed nearly on all MM tumor cell lines (80%–100%) (9), and the quantity of BCMA on the surface of malignant PCs is much higher than regular PCs (10). The ligands of BCMA include BAFF and a proliferation-inducing ligand (APRIL), which is a homolog of BAFF (11). APRIL has a higher affinity for BCMA than BAFF (12), and both of them can activate the downstream signals of BCMA like nuclear factor kappa-B (NF-κB) (13), rat sarcoma/mitogen-activated protein kinase (RAS/MAPK), and phosphoinositide-3-kinase–protein kinase B/Akt (PI3K-PKB/Akt) (14), thus promoting the expression of antiapoptotic proteins (e.g., Mcl-1, BCL-2, BCL-XL) and the activation of specific signaling pathways or factors (e.g., cell adhesion molecules, angiogenesis factors, immunosuppressive molecules) about cells’ proliferation (14). One of these factors is c-Jun N-terminal kinase (JNK) (7), which can work together with NF-κB, JAK/STAT, and other related signaling molecules to synergistically promote tumor cell survival in the tumor microenvironment (TEM) (15). A study reported in ASH2021 (16) found that the expression of SETD2 can activate the BCMA-JNK pathway, thus facilitating the proliferation and maintenance of myeloma cells. Bridging this gap is the regulation of H3K36 trimethylation (H3K36me3) by SETD2, which provides us with a new perspective to explain the upstream activation of BCMA and the stimulation of its downstream signal pathways through epigenetic mechanisms. It is worth mentioning that an increasing number of studies have confirmed the critical role of epigenetics in MM. For instance, the overexpression of histone methyltransferase MMSET can stimulate H3K36me2, which has been identified as one of the pathogenic mechanisms of t(4;14)^+^ MM (17). The membrane-bound BCMA can break off from the cell membrane by the shear function of γ-secretase and turn into a soluble BCMA (sBCMA) (18), which is closely related to the development of MM and the prognosis of patients (19, 20). The formation of sBCMA reduces the distribution of BCMA on tumor cells’ surface, thus relieving the effect conducted by BCMA activation. However, this mechanism may lower the efficacy of BCMA-targeted immunotherapies as well, resulting in MM cells’ immune escape. With the gradual deepening of our awareness about the underlying mechanism related to BCMA, the modification to multiple existing anti-BCMA immunotherapies is also accelerating its pace.

## 3 Progress of BCMA-Targeted Immunotherapies

### 3.1 BCMA-Targeted mAbs

The finding of BCMA-targeted mAbs can be regarded as an essential milestone in the field of targeted immunotherapy for MM. The first two approved mAbs agents target CD38 antigen (daratumumab) (3) and signaling lymphocytic activation molecule family member 7 (SLAMF7) (elotuzumab) (4), respectively. Although their effectiveness has been proved, there are still a large number of patients who relapse after receiving more than 3 prior lines of therapy (LOT), the mAbs, and further progress to relapsed or refractory multiple myeloma (RRMM) (21). At present, along with myriad novel immunotherapy agents being developed, researchers are also looking for more mAbs that can work better. SEA-BCMA, a novel humanized nonfucosylated IgG1 mAb, targets BCMA, which is expressed on the malignant PCs. The working mechanisms of SEA-BCMA may include blocking of BCMA activation with its downstream proliferative signaling pathways, regulating antibody-dependent cellular phagocytosis effect, and reinforcing the antibody-dependent cellular cytotoxicity. ASH2021 updated some findings regarding this agent (22, 23).

The preliminary results of its phase I clinical trial (SGNBCMA-001; NCT03582033) (23) are reported in this meeting. Part A of SGNBCMA-001 conducted a dose-escalation trial (from 100 to 1,600 mg, Q2W) of SEA-BCMA monotherapy for RRMM patients without any prior treatments. At the 800-mg Q2W regimen, 1 of 7 patients reported a grade 3 infusion-related reaction (IRR), which was the single dose-limiting toxicity (DLT) observed during dose escalation. At the maximum dose (1,600 mg Q2W, *n* = 22), the objective response rate (ORR) was 14% (*n* = 3). One patient got very good partial responses (VGPR), and two got partial responses (PR). The adverse events (AEs) were fatigue (32%), pyrexia (23%), IRR (23%), hypertension (23%) unrelated to hematological incidents, and anemia (14%) related to hematologic incidents from high to low. The other parts of this trial designed to verify whether using SEA-BCMA in higher doses (part B, Q1W induction dosing of SEA-BCMA for 8 weeks is followed by Q2W maintenance dosing) or combining it with dexamethasone (DEX) (part C) can produce better therapeutic results for the patients who have received ≥3 prior LOT for MM and were triple-class refractory. Surprisingly, DLTs did not occur in these two parts. Two of eight (2 PR) and two of twelve (1 VGPR, 1 PR) patients reported a certain OR in parts B and C, respectively. The pharmacokinetics (PK) analysis showed that the half-life of SEA-BCMA was approximately 10 days, and either ascending dose (from Q2W to Q1W) or combining DEX had no significant effect on its metabolism.

Moreover, Taft et al. reported the binding and saturation pharmacodynamics (PD) of SEA-BCMA in patients enrolled in part A of SGNBCMA-001. They suggested that the sBCMA in plasma may affect tumor cell clearance because of the formation of sBCMA : SEA-BCMA complex. Interestingly, an amplification dose of 1,600 mg seems to overcome this negative effect and support malignant plasma cell drug exposure. Nevertheless, the dose dependence of SEA-BCMA needs a more comprehensive evaluation. These latest results proved the safety of SEA-BCMA and the possibility to combine it with other medicines for patients with MM. Further studies will carry out in the subsequent part D, and it is expected to be used into clinical application as a promising anti-BCMA agent.

### 3.2 Bispecific Antibodies

Bispecific antibodies (BsAbs), which have affinities for two different epitopes on tumor cells and specific immune cells like T cells, as a bridge, induce the formation of immunological synapses between T cells and tumor cells, which can make granular enzymes and perforin released by T cells produce lethal effect to the targeted tumor cells (24). Up to now, BCMA, CD38, and SLAMF7 have been selected as the targets to prepare BsAbs for the treatment of MM (25). BiTE, a special BsAb, can physically bind BCMA and CD3ε on T-cell receptors (TCR) for redirecting T cells to myeloma cells to exert its cytotoxicity (25). Also, many new targets have been identified, like G-protein coupled receptor C family 5D (GPRC5D) (26), which are also expressed highly on the surface of PCs (27). ASH2021 provided us with the latest data from the early-stage clinical trials of multiple novel BsAbs for treating MM, which could certify the efficacy and safety of these new agents (**Table 1**).

**Table 1:** Updated clinical data for BsAbs.

		RO7297089-GO4158 (NCT04434469)	Teclistamab (JNJ-64007957)-MajesTEC-1 (NCT04557098)		REGN5458-(NCT03761108)		Tnb-383B-(NCT03933735)	Elranatamab (PF-06863135)–MagnetisMM-1 (NCT03269136)	
Phase		1	1/2		1/2		1	1	
Structure		BCMA×CD16a (BsAbs)	BCMA×CD3 (BsAbs)		BCMA×CD3 (BsAbs)		BCMA×CD3 (BsAbs)	BCMA×CD3 (BiTEs molecule)	
Schedule		Dose escalation: 60, 180, 360, 1,080, 1,850 mg	1,500 µg/kg/w followed by step-up doses of 60 and 300 µg/kg		Dose escalation: full doses ranging from 3 to 400 mg		Dose escalation/expansion: 0.025–120 mg	Part 1: 80, 130, 215, 360, 600, and 1,000 μg/kg/w (SC)	
								Part 1.1/2A (RP2D): single priming dose (600 μg/kg or equivalent fixed dose of 44 mg), then the full dose (1,000 μg/kg or equivalent fixed dose of 76 mg) Q1W or Q2W followed (SC)	
								Part 1C/1D: single priming dose (32 mg), then the full dose (44 mg) Q1W followed one week later in combination with either LEN (25 mg) or POM (4 mg) on days 1 to 21 of a 28-day cycle (SC)	
Patients (*n*)		21	159 (phase 1: *n* = 40; phase 2: *n* = 119)		68		103 (dose escalation: *n* = 73; dose expansion: *n* = 30)	58 (part 1.1: *n* = 50; part 1C: *n* = 4; part 1D: *n* = 4)	
Efficacy									
ORR (%)	NA		65 (phase 1 pts)	73.3 (96 and 200 mg dose levels)		79 (19/24) (≥40 mg dose-escalation cohort); 64 (28/44) (≥40 mg dose-escalation and dose-expansion cohorts)			70 (14/20) (part 1, at the efficacious dose range 215–1,000 μg/kg)
≥CR rate (%)	NA		40 (phase 1 pts)	19.1 (13/68) (across all dose levels)		29 (7/24) (≥40 mg dose-escalation cohort); 16 (7/44) (≥40 mg dose-escalation and dose-expansion cohorts)			30 (6/20) (part 1, at the efficacious dose range 215–1,000 μg/kg)
≥VGPR rate (%)	NA		60 (phase 1 pts)	36.8 (25/68) (across all dose levels)		63 (15/24) (≥40 mg dose-escalation cohort); 43 (19/44) (≥40 mg dose-escalation and dose-expansion cohorts)			35% (7/20) (part 1, at the efficacious dose range 215–1,000 μg/kg)
Safety									
Nonhematologic TRAEs	IRR (48%); back pain (24%); ALT rise (19%)		CRS (67%); injection site erythema (23%); fatigue (22%); ICNS (4 pts)	CRS (38.2%); fatigue (20.6%)		77% (Gr ≥3:32%, serious AEs:22%): CRS (52%); neutropenia (17%); fatigue (14%)			
Hematologic TRAEs	Anemia (52%); thrombocytopenia (19%)		Neutropenia (53%); anemia (41%); thrombocytopenia (33%)	Neutropenia (16.2%)					
TEAEs				97.1% (≥Gr3: 76.5%): fatigue (42.6%); CRS (38.2%); nausea (32.4%)		8%: infections (28%); pneumonia (5%)			CRS (83%); lymphopenia (64%); neutropenia (64%); anemia (55%); injection site reaction (53%); thrombocytopenia (52%)
Reference	(28)		(29)	(30)		(31)			(32)

BCMA, B-cell maturation antigen; BsAbs, bispecific antibodies; BiTEs, bispecific T-cell engagers; w, week; LEN, lenalidomide; POM, pomalidomide; ORR, overall response rate; NA, not applicable; CR, complete response; VGPR, very good partial response; DOR, duration of response; TRAEs, treatment-related AEs; IRR, infusion-related reaction; CRS, cytokine release syndrome; pts, patients; Gr, grade; AEs, adverse events; ICANS, immune effector cell-associated neurotoxicity syndrome; TEAEs, treatment-emergent AEs; DLT, dose-limiting toxicity; SC, subcutaneous.

Some information about **Table 1** should be added: firstly, the target CD16a of RO7297089 is expressed on the innate immune cells such as monocyte subsets, macrophages, and natural killer (NK) cells. Among the five dose cohorts in this study, ten patients had stable disease as their best response at dose levels of 60 mg (1/3 patients), 180 mg (2/5 patients), 360 mg (3/4 patients), and 1,080 mg (4/6 patients). Its PK parameter was nonlinear (a more than dose proportional increase) as the doses of RO7297089 increased from 60 to 1,080 mg, and then approached linear at doses higher than 1,080 mg. The disposition of this agent was mediated by its target. Secondly, the phase I study of teclistamab has obtained its recommended phase II dose (RP2D), which was applied in the phase II study. The data on the effectiveness of this drug in **Table 1** showed how the 40 patients who participated in phase I (median follow-up: 6.1 months) performed in phase II (median follow-up: 8.2 months), which was consistent with previously presented data (65% ORR and 58% VGPR rate) in phase I study. Thirdly, the median follow-up duration of the patients enrolled in REGN5458 clinical trial was 2.4 months. Although median DOR was not reached in this trial, the probability of DOR ≥8 months was 92.1%. Fourthly, the RP2D of Tnb-383B was 60 mg Q3W. The median follow-up time of the ≥40mg dose-escalation cohorts and the ≥40mg combined dose-escalation and dose-expansion cohorts were 6.1 and 3.1 months, respectively. Fifthly, elranatamab is a humanized bispecific molecule. Its subcutaneous (SC) cohorts from MagnetisMM-1 contained five parts: dose escalation (part 1), monotherapy with priming (part 1.1), lenalidomide (LEN) combination (part 1C), pomalidomide (POM) combination (part 1D), and monotherapy expansion with priming (part 2A). In part 1, the efficacious dose range was 215–1,000 μg/kg. ASH2021 updated the ORR and sCR/CR rate under these doses, and the confirmed ORR at the RP2D was 83% (5/6) in this part. One last thing worth mentioning is that, although the patients enrolled in the REGN5458 clinical trial were penta-refractory after 5 or so prior LOT, the rest of the patients enrolled in the other trials relapsed after ≥3 prior LOT including a proteasome inhibitor, an immunomodulatory drug, and a CD38-targeted therapy.

### 3.3 Novel BCMA-Targeted Tri-Specific Agents

Frankly speaking, today’s researchers are no longer satisfied with the dual-target immunotherapies for treating the patients with RRMM. The current studies have reached a level of developing triple or multiple specificity agents, which may have better efficacy. ASH2021 reported that HPN217, a half-life extended (median serum half-life: 74 h) (33) tri-specific T-cell activation construct (TriTAC) synchronously targeting BCMA, serum albumin to prolong the half-life period, and CD3ε to active and redirect T cells, could exert their cytotoxic effect to myeloma cells (34). The preclinical translational studies showed that HPN217 could eliminate 71% of tumor cells at a 0.45-T cell/MM cell ratio. The density of BCMA and the sBCMA in circulation affected the tumor killing effect of HPN217. Consistent with this result, GSI (e.g., LY-3039478), which increased the expression of BCMA on the surface of myeloma cells, could enhance the efficacy of this agent. Moreover, the negative effect of DEX on the HPN217-redirected T cells may be restricted (34). The phase I clinical trial is ongoing, whose preliminary results showed us that the maximum safe dose of HPN217 was 2,150 µg/week and its treatment-emergent AEs were transient and controllable (33). Another BCMA-targeted tri-specific agent who was undergoing preclinical evaluation has been reported in this meeting as well (35). CDR101, targeting CD3, BCMA, and PD-L1, could guide T cells to BCMA-expressed tumor cells and play a role in combating immunosuppression caused by the interaction of PD-L1 and PD-1 at the immune synapse site, which may reduce the possibility of “on-target off-tumor” effects. Compared with BCMA × CD3 bispecifics, CDR101 resulted in at least 10-fold increased T-cell-mediated tumor cells lysis and it performed better than the combination of the PD-L1 inhibitors and BCMA × CD3 bispecifics. Based on these findings, it is suggested that novel tri-specific immunotherapy agents argue for a high clinical potential and promising translation into the clinic.

### 3.4 Antibody-Drug Conjugates

Antibody-drug conjugates (ADCs), which connected mAbs with bioactive drugs through chemical linkers (36), can accurately identify tumor cells and exert high-efficiency cytotoxic effects on malignant cells without damaging healthy tissues (37). Belantamab mafodotin (GSK2857916), which is a microtubule-disrupt agent (38), consists of humanized BCMA-targeted IgG1 and monomethyl auristatin-F (MMAF). Blenrep was approved by the U.S. Food and Drug Administration (FDA) in 2020 for treating patients with RRMM. As the first licensed BCMA-targeted immunotherapy for marketing (39), belantamab has been tested in multiple clinical trials (38–40), which could confirm its safety and efficacy. The first-in-human DREAMM-1 study showed that the belantamab monotherapy (3.4 mg/kg, Q3W) induced deep (overall response: 60%, 21/35) and durable (median DOR: 14.3 months) responses (38). The results of DREAMM-2 (NCT03525678), a multicentric phase II clinical study of this ADC, have confirmed that the recommended regimen for its future studies was 2.5 mg/kg, Q3W instead of 3.4 mg/kg, Q3W, which was the RP2D after the phase I trial. Under this dose, the ORR was 31% (30/97) with manageable safety profile (41). Even so, belantamab also has a certain extent of boundedness. DREAMM-2 demonstrated that the toxicity of this agent was mainly reflected in thrombocytopenia and lesions about the cornea, which presented as microcyst-like epithelial changes or superficial punctate keratopathy (41). Moreover, adverse ocular signs like dry eye and diminution of best-corrected visual acuity (BCVA) have occurred during the administration of belantamab as well (41). Given that changing its administration regimens may reduce the incidence of corneal events without compromising the therapeutic effect, a new phase II, 5-arm, open-label and multicentric clinical trial DREAMM-14 is preparing to determine if there are better dosage choices than 2.5 mg/kg Q3W. This study will initiate in the springtime of 2022 (42). To relieve stress in the real world, a study in ASH2021 analyzed if those relatively simple clinical indicators or convenient judgment methods such as questionnaires, could replace the professional eye examinations for determining whether to change the in-use medication regiments (43). The conclusion of this study was unequivocally positive, and once these strategies are applied in clinical practice, the burden of either patients or physicians will greatly reduce. DREAMM-1 and DREAMM-2 have studied the efficacy of belantamab monotherapy. ASH2021 updated the results of belantamab/DEX and belantamab/DEX + POM for the patients with triple-class refractory disease. After the combination of belantamab (2.5 mg/kg Q3W) with DEX (20–40 mg Q1W, median 3 cycles), the ORR was 46%, the CR rate was 14%, and 18% of all patients achieved ≥VGPR with 7.4 months median follow-up duration. Median progression-free survival (PFS) was 4.9 months, with 7.4 months’ median overall survival (OS). The incidence of AEs were anemia (83%), keratopathy (82%; Gr3/4: 56%), thrombocytopenia (70%), neutropenia (30%), and elevated liver function tests (53%) from high to low (44). On the other hand, after the combined application of belantamab (1.92, 2.5, or 3.4 mg/kg, Q4W, designed by 3 + 3 dose escalation strategy), POM (an IMiD) (4 mg day 21/28 days), and DEX (40/20 mg weekly), the ORR was 88.9% (48/54) and the sCR, ≥VGPR, and PR rates were 24.1% (13/54), 68.5% (37/54), and 20.4% (11/54), respectively. The median PFS was 24.2 months based on a median of 8.6 months follow-up. Keratopathy (96.9%) also was the most common AEs, and 56.7% of such patients have reached Gr 3/4 (45). These two studies demonstrated that POM and DEX may have positive impacts on the efficacy of belantamab. However, keratopathy remains a challenge in the treatment process. In addition to belantamab mafodotin, several other BCMA-targeted ADCs, such as AMG 224, MEDI2228, and HDP-101, are also undergoing multiple preclinical or clinical studies in different phases.

### 3.5 BCMA-Targeted CAR-T-Cell Therapy

Compared with the mAbs, BsAbs, and ADCs mentioned above, the therapeutic effect for BCMA-targeted CAR-T-cell therapy presented in the 63rd ASH seems to be more optimistic. Moreover, the preliminary results of many other related studies, such as the engineering improvement strategies to existing CAR-T-cell products or the effects induced by multiple factors inside and outside the body, were reported in this meeting. The relevant data about the safety and efficacy of those products, including ciltacabtagene autoleucel (cilta-cel) (46–48), CT053 (49, 50), CT103A (51), C-CAR088 (52), PHE885 (53), CART-ddBCMA (54), and bb21217 (55), are presented in **Table 2**, and the relevant supplementary explanations will be carried out later in this paper.

**Table 2:** BCMA-targeted CAR-T cells in clinical trials.

Name (manufacturer)	Clinical trial information	Inclusion/exclusion criteria		Pt characteristics	Dosage	Major response	Most common AE
Ciltacabtagene autoleucel (Janssen, Xi'an, China)	Phase 1b/2 (NCT03548207) (46)	RRMM who received or were refractory to ≥3 prior lines, including PI, IMiD, CD38 mAb		97 pts; median age 61; median prior lines 6	Single cilta-cel infusion (target dose 0.75 × 10^6^ CAR^+^ viable T cells/kg; range 0.5–1.0 × 10^6^) 5–7 days after lymphodepletion (300 mg/m^2^ cyclophosphamide, 30 mg/m^2^ fludarabine daily for 3 days)	ORR 97.9%; sCR 80.4%; VGPR 14.4%; PR 3.1%; NR 2.1%	G3-4 neutropenia (94.8%), anemia (68.0%), leukopenia (60.8%), thrombocytopenia (59.8%), lymphopenia (49.5%); CRS (94.8%); neurotoxicity (0%)
	Phase 2 (NCT04133636) (47, 48)	Cohort A (47)	RRMM who received or were refractory to ≥3 prior lines, including PI, IMiD, CD38 mAb, lenalidomide relapse; hx of BCMA-directed therapy were excluded	20 pts; median age 60; median prior lines 2	Single cilta-cel infusion (target dose 0.75 × 10^6^ CAR^+^ viable T cells/kg) 5–7 days after lymphodepletion (300 mg/m^2^ cyclophosphamide, 30 mg/m^2^ fludarabine daily for 3 days)	ORR 95%; CR 85%; VGPR 10%	G3-4 neutropenia (95%), thrombocytopenia (35%), anemia (45%), lymphopenia (60%), leukopenia (55%); CRS (95%), G3-4 CRS (10%); G1-2 neurotoxicity (20%)
		Cohort B (48)	RRMM who received or were refractory to 1 prior line, including PI, IMiD, had disease progression either ≤12 months after ASCT or ≤12 months after start of antimyeloma therapy except ASCT, were tx-naïve to CAR-T or anti-BCMA therapies	18 pts; median age 57	Single cilta-cel infusion (target dose 0.75 × 10^6^ CAR^+^ viable T cells/kg) 5–7 days after lymphodepletion (300 mg/m^2^ cyclophosphamide, 30 mg/m^2^ fludarabine daily for 3 days)	ORR 100%; CR 31.2%; VGPR 43.8%; PR 25%	Neutropenia (88.9%), thrombocytopenia (61.1%), anemia (50.0%), leukopenia (27.8%), and lymphopenia (22.2%); G1-4 CRS (83.3%); G1 neurotoxicity (5.6%)
CT053 (CARsgen, Shanghai, China)	Phase 1 (NCT03975907) (NCT03380039, NCT03716856, NCT03302403) (49, 50)	RRMM who received or were refractory to ≥2 prior lines, including PI, IMiD, CD38 mAb		38 pts	0.5 (*n* = 1), 1.0 (*n* = 4), 1.5 (*n* = 32), 1.8 (*n* = 1) × 10^8^ CAR^+^ viable T-cell infusion after lymphodepletion	ORR 92.1%; CR 78.9%; VGPR 7.9%; PR 5.3%; NR 7.9%	G1-2 CRS (73.7%); G3 neurotoxicity (0%); DLT (0%)
CT103A (Sana, Seattle, USA) (IASO, Nanjing,China)	Phase 1/2 (NCT05066646) (51)	RRMM who received or were refractory to ≥3 prior lines, including PI, IMiD, CD38 mAb		71 pts; median age 58; median prior lines 4	1.0 × 10^6^ CAR^+^ viable T cells/kg single infusion 1 d after lymphodepletion (300 mg/m^2^ cyclophosphamide, 30 mg/m^2^ fludarabine daily for 3 days)	ORR 94.4%; CR 50.7%; VGPR 26.8%; PR 16.9%	CRS (93%), G3 CRS (2.8%); G2 neurotoxicity (1.4%)
C-CAR088 (CBMG, Delaware, USA)	Phase 1 (NCT04295018, NCT04322292, NCT03815383, NCT03751293) (52)	RRMM who received or were refractory to ≥2 prior lines, including PI, IMiD, CD38 mAb		31 pts; median age 61; median prior lines 4	1.0, 3.0, 4.5~6.0 × 10^6^ CAR^+^ viable T cells/kg infusion after lymphodepletion (300 mg/m^2^ cyclophosphamide, 30 mg/m^2^ fludarabine daily for 3 days)	ORR 96.4%; CR 57.2%; VGPR 32.1%; PR 7.1%	CRS (93.5%), G1 CRS (58.1%), G2 CRS (25.8%), G3 CRS (9.7%); neurotoxicity (3.2%)
PHE885 (Novartis, Basel, Switzerland)	Phase 1 (NCT04318327) (53)	RRMM who received or were refractory to ≥2 prior lines, including PI, IMiD, CD38 mAb		6 pts; median prior lines 5	5.0, 14.3 × 10^6^ CAR^+^ viable T cells/kg infusion after lymphodepletion	ORR 100%; CR 17%; VGPR 33%; PR 50%	≥G3 anemia (100%), neutropenia (100%), thrombocytopenia (67%), leukopenia (33%), ALT and AST increase (33%), decreased blood fibrinogen (33%); CRS (33%); G3 CRS (100%); G2 neurotoxicity (33.3%)
CART-ddBCMA (Arcellx, Maryland, USA)	Phase 1 (NCT04155749) (54)	RRMM who received or were refractory to ≥3 prior lines, including PI, IMiD, CD38 mAb		16 pts; median age 66; median prior lines 5	100, 300 × 106 ( ± 20%) CAR^+^ viable T cells/kg infusion after lymphodepletion (300 mg/m^2^ cyclophosphamide, 30 mg/m^2^ fludarabine daily for 3 days)	ORR 100%; sCR 43.8%; CR 12.5%; VGPR 18.7%; PR 25%	CRS (100%); ≥G3 CRS (6%); G3 neurotoxicity (13%)
bb21217 (bluebird bio, Massachusetts, USA)	Phase 1 (NCT03274219) (55)	RRMM who received or were refractory to ≥3 prior lines, including PI, IMiD, CD38 mAb		72 pts	150, 300, 450 × 10^6^ CAR^+^ viable T cells/kg infusion after lymphodepletion (300 mg/m^2^ cyclophosphamide, 30 mg/m^2^ fludarabine daily for 3 days)	ORR 69%; CR 28%; VGPR 30%; PR 11%	CRS (75%); G1-2 CRS (70.8%)
							G3 CRS (1.4%); neurotoxicity (15%)

Pt, patient; AE, adverse event; ORR, overall response rate; sCR, strict complete response; VGPR, very good partial response; PR, partial response; NR, no response; G, grade; CRS, cytokine release syndrome; hx, history; CR, complete response; mo, month; ASCT, autologous stem cell transplantation; tx, treatment; DLT, dose-limiting toxicity.

#### 3.5.1 Ciltacabtagene Autoleucel

Cilta-cel, one of the BCMA-targeted CAR-T-cell products with two anti-BCMA single-domain antibodies to present avidity, a CD3-ζ signaling domain, and a 4-1BB costimulatory domain (56), has gotten favorable responses in its phase Ib/II open-label study CARTITUDE-1 from 97 patients with MM who had relapsed after more than three prior LOTs, such as PI, IMiD, or MoABs (56). According to the past report, after 5–7 days of single cilta-cel infusion (0.75 × 10^6^ cells/kg) and median 12.4 months of follow-up, the ORR was 97%, with 67% of patients achieving sCR. Twelve months PFS rate and OS rate were 77% and 89%, respectively. There were two reports (46, 57) in ASH2021 presented the subsequent results of these patients and the performance of the subgroups in CARTITUDE-1. After 18 months median follow-up, the resulting ORR was 97.9%, which was well-matched to all the subgroups. 80.4% of all subjects got sCR, and 94.8% achieved VGPR or better. The median DOR was 21.8 months. The rate of 18 months’ PFS and OS was 66.0% and 80.9%, respectively. These data also were consistent with most of the subcohorts. As for minimal residual disease (MRD), 91.8% of those who had been tested (*n* = 61) reported MRD negative at the 10^−5^ threshold. Across all the subgroups, the data were 80% to 100%. In terms of the safety, the mainly hematologic AEs graded 3 or 4 were neutropenia (94.8%), anemia (68.0%), leukopenia (60.8%), thrombocytopenia (59.8%), and lymphopenia (49.5%), without cytopenia-related fatalities. 94.8% of all the patients occurred CRS, and 98.9% of them obtained remission within 14 days. No neurotoxicity case related to CAR-T cells happened since the last report. The efficacy and safety of cilta-cel can be proved by the results from this phase Ib/II study, and the comparison between cilta-cel and other kinds of therapeutic methods for MM can extend its advantages to real-world clinical practice (RWCP). LocoMMotion (58), which can be seen as an external control cohort of CARTITUDE-1, is the first prospective study for cilta-cel’s applicability in the real world (59). In LocoMMotion, 246 patients with RRMM who relapsed after more than triple class exposure to IMiDs, PIs, and MoABs were enrolled, they then received more than ninety other treatment regimens besides CAR-T-cell therapy. Based on the comparative analysis of many aspects between LocoMMotion and CARTITUDE-1, the prognosis of the patients treated with other therapeutic strategies was worse. Cilta-cel had a better outcome reflected by many indicators including ORR, CR, PFS, and OS.

CARTITUDE-2, a phase II multicohort clinical trial for cilta-cel, is currently ongoing. Two reports in ASH2021 provided us with the updates of cohort A (47) and cohort B (48) in CARTITUDE-2, respectively. In cohort A, 20 patients who were refractory after more than three prior LOTs especially lenalidomide were treated with cilta-cel (0.75 × 10^6^ cells/kg, 5–7 days). The ORR was 95%; 85% of patients performed better than complete response (CR), and 95% of them were superior to VGPR. Median DOR has not been reached, but the 6-month PFS rate was 90%. In total, 13 patients were evaluated for MDR, and 92.3% of them got MRD negative based on the 10^−5^ criterion. The common hematologic AEs were neutropenia (95%), thrombocytopenia (80%), anemia (75%), lymphopenia (65%), and leukopenia (55%). Although the incidence rate of CRS was 95%, 90% of these cases were cured within 7 days. This, together with the neurotoxicity that happened in only 20% of all patients, demonstrated the manageable safety profile of cilta-cel. In cohort B, the cilta-cel infusion (0.75 × 10^6^ cells/kg, 5–7 days) performed in 18 patients who relapsed within 12 months after receiving autologous stem cell transplantation or other anti-MM therapies. After an average of 4.7 months’ follow-up, the ORR reached 100%. In total, 31.2% of them achieved better than CR, and 75% were superior to VGPR. All the evaluated patients (*n* = 9) performed MRD negative. With 4 days median time of duration (ranged 1–7), CRS (grades 1–4) occurred in 83.3% of patients, and ICANS (grade 1) occurred in only one patient. For cilta-cel, the latest results of CARTITUDE-1 and CARTITUDE-2 jointly highlight its potential as a promising method for heavily pretreated patients with RRMM. Further studies including CARTITUDE-4 (NCT04181827) have been carried out. However, this agent has not been approved yet for marketing.

#### 3.5.2 Idecabtagene Vicleucel (ide-cel, bb2121)

Based on the positive results from the pivotal single-arm, open-label phase II clinical trial called KarMMa (60), Abecma (ide-cel), one of the BCMA-targeted CAR-T-cell products, which is used to treat the patients with RRMM after four or more prior LOTs including IMiD, PI, and MoABs (61), has been approved for listing by FDA as the first one around the world. ASH2021 updated the study results of health-related quality of life (HRQoL) in KarMMa (62). The results which have been reported previously have shown the significant clinical benefits of ide-cel on HRQoL during a 9-month follow-up (63), and the updated performance of the patients enrolled in this trial also proved that after 24 months follow-up, notable HRQoL improvements in multiple predefined domains were achieved. In those predefined prime HRQoL domains, 40%–70% of all 128 patients had clinically meaningful advances reflected by many indicators, such as QLQ-C30 fatigue, pain, physical functioning, and global health status/QoL scores at the later time points. In addition, 30%–40% of these patients got improvements in cognitive function, disease symptoms, and side effects, with 40%–60% of them remaining stable in these domains. Among those predefined secondary HRQoL domains, the improvement in role functioning, emotional functioning, social functioning, dyspnea, insomnia, constipation, diarrhea (QLQ-C30), future perspectives (QLQ-MY20), health utility index scores (EQ-5D-5L), and VAS scores (EQ-5D) had clinical significance. It is worth mentioning that there was also a study in ASH2021 (64), which was a qualitative analysis of the interviews with patients in KarMMa after 6–24 months ide-cel treatment, provided us a novel insight to evaluate the posttreatment life quality by analyzing the attitude of patients. Undoubtedly, 73% of all interviewed subjects (*n* = 33) had positive attitude towards ide-cel infusion.

Moreover, ASH2021 also touched on some of other studies derived from KarMMa. Because of the difference in overall OS and median PFS (34.2, 24.8 months and 8.8, 8.6 months, respectively) between the results of KarMMa and an earlier phase I study of ide-cel named CRB-401 (65), further research was conducted on the patients enrolled in KarMMa who relapsed after ide-cel treatment. A report (66) showed us the difference between those who received subsequent antimyeloma therapy (sAMT) (*n* = 68) and the anti-BCMA therapy (*n* = 11) after ide-cel infusion: the median PFS and OS of the patients with sAMT were 6.1 and 24.8 months, respectively. The duration of overall sAMT was 215 days, and the second disease progression (PFS2) was 13.6 months (inclusive of time on ide-cel therapy). The median PFS and OS of the patients who were applied anti-BCMA therapy was 12.1 and 31.0 months, and the median duration of the first sAMT was 48 days with 15.5 months’ PFS2, which was more favorable. Therefore, patients who relapsed after the first ide-cel infusion may benefit from the follow-up anti-BCMA therapy, while the emergence of this phenomenon requires conditions referred to a past study (67). There was also a report (68) about the infectious complications after ide-cel treatment in patients with RRMM from CRB-401 and KarMMa. The overall incidence of infection matched the previous data of CD19 CAR-T-cell therapy. Generally, bacterial infections were the most common, and only one patient developed fungal infection despite none of the patients receiving antifungal prophylaxis. This study provided us with some other explicit information on specific infectious complications of the particular crowd, and it was pregnant to the clinical application of ide-cel.

#### 3.5.3 Updated Information of Other Existing BCMA-Targeted CAR-T Products

**Figure 2:**
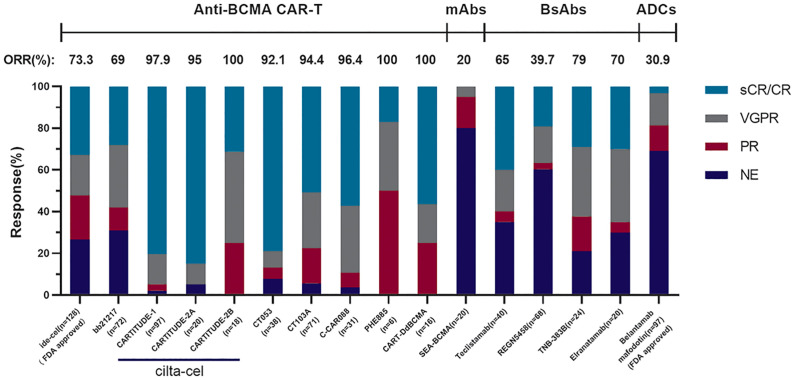
The comparison of different anti-BCMA agents. These results come from phase II clinical trial KarMMa of Idecabtagene Vicleucel (*n* = 128, 13.3 months median follow-up), phase I clinical trial CRB-402 of bb21217 (*n* = 72, 9 months median follow-up), phase Ib/II clinical trial CARTITUDE-1 of cilta-cel (*n* = 97, 18 months median follow-up), cohort A in phase II clinical trial CARTITUDE-2 of cilta-cel (*n* = 20, 9.7 months median follow-up), cohort B in phase II clinical trial CARTITUDE-2 of cilta-cel (*n* = 18, 4.7 months median follow-up), phase I/II clinical trial LUMMICAR STUDY 1 of CT053 (*n* = 14, 13.6 months median follow-up), phase I/II clinical trial of CT103A (*n* = 71, 147 days median follow-up), phase I clinical trial of C-CAR088 (*n* = 31, 8 months median follow-up), phase I clinical trial of PHE885 (*n* = 6, 1 month follow-up), phase I clinical trial of CART-ddBCMA (*n* = 16, 155 days median follow-up), phase I clinical trial SGNBCMA-001 of SEA-BCMA (*n* = 20, 12 weeks median follow-up), phase II clinical trial MajesTEC-1 of teclistamab (*n* = 40, 8.2 months median follow-up), phase I clinical trial of REGN5458 (*n* = 68, 2.4 months median follow-up), the dose-escalation cohorts in phase I clinical trial of TNB-383B (*n* = 24, 6.1 months median follow-up), and the patients treated across the efficacious dose range (215–1,000 μg/kg) in part 1 of phase I clinical trial MagnetisMM-1 of elranatamab (*n* = 20, 22 days median follow-up).

In addition to cilta-cel and ide-cel, a variety of other BCMA-targeted CAR-T-cell products were mentioned at this ASH meeting. The relevant data are presented in **Table 2**, and there are some points that should be added: firstly, CT053, an all-human CAR-T-cell product, has shown promising efficacy and safety in its phase I clinical trial (LUMMICAR STUDY 1 and CG) (49, 50). Two things interesting were that, the ORR of CT053 to treat the RRMM patients relapsed after three or more LOT with the extramedullary disease (EMD) being 91.7%, the CR rate being 58.3%, and the median PFS being 9.3 months, better than the results of those past treatment strategies such as combination therapy with permadomide and dexamethasone (ORR: 30%; CR rate: 15.3%) (69) and carfilzomib-based combination therapy (ORR: 27%, CR rate: 0%, median PFS: 5 months) (70). For the patients with high-risk cytogenetic abnormalities [del(17p), t(4;14), t(14;16)/1q21], the ORR and CR rate of CT053 were 84.2% and 73.3%, respectively, with the 15.6 months median PFS, better than the results of ishatuximab, permadomide, and dexamethasone combination (ORR: 50%, CR rate: 0%, median PFS: 7.5 m) (71), carfilzomib monotherapy (ORR: 25.8%, CR rate: 0%, median PFS: 3.5 m) (72), and even the infusion of bb2121 (ORR: 73%, CR rate: 33%, median PFS: 8.2 m) (60). Secondly, the relationship between the dosage and curative effect of C-CAR088 has been studied (52). Among the selected doses of 1.0, 3.0, and 4.5~6.0 × 10^6^ CAR^+^ T cells/kg, the cohorts whose dosage ≥3.0 × 10^6^ CAR-T cells/kg had deeper and more durable responses, which needed further research. Thirdly, PHE885 is a novel fully human CAR-T-cell product modified with T-Charge™. This platform can reduce the *in vitro* culture time of CAR-T cells to about 24-h, thus taking only less than 2 days to acquire the final products, which totally depends on the *in vivo* proliferation after infusing CAR-T cells (53). The application of this new platform also can retain the naïve-like and stem cell memory T cells (Tnaïve^+^Tscm) (CD45RO^−^/CCR7^+^), which are beneficial to the persistence of CAR-T cells. By contrast, the CAR-T cells (TM_PHE885), having the same single-chain variable fragments (scFvs) of PHE885, which were prepared by the conventional methods, just keeps central-memory T cells (CD45RO^+^/CCR7^+^) (73). Strong cell amplification was observed in all patients by qPCR technique (the maximum amplification of T cells in circulation was 283,000 copies/μg, the median maximum amplification time was 21.1 days) and flow cytometry (the maximum amplification of T cells in circulation was 69.3%, the median maximum amplification time was 16.4 days). PHE885 can be detected in the peripheral blood of each patient during follow-up (1–6 months). Fourthly, CART-ddBCMA is a special CAR-T-cell product with a synthetic BCMA binding domain. Differing from the classical scFvs with a 4-1BB costimulatory domain and a CD3ζ activation domain, it is a smaller stable protein containing only 73 amino acids, thus reducing the threats of immunogenicity (54). Finally, a multicenter phase I trial of bb21217 named CRB-402 (NCT03274219) is underway. The preliminary results of its preclinical study have been reported before, and the subsequent results presented in ASH2021 showed that adding PI3K inhibitor (BB007) during the *in vitro* culture stage to amplify memory-like T cells (CD62L^+^ and CD27^+^) (74, 75) could advance the persistence of CAR-T cells literally. This positive effect was reflected on a better DOR of bb21217 compared with bb2121 (65), which shared the same CAR structure with it (55). Moreover, after infusing bb21217, CAR-T cells could be detected in 30/37 (81%) patients and 9/15 (60%) patients at 6th and 12th months. Analysis of the peripheral blood samples showed that less differentiated, more proliferative CAR-T cells at peak expansion are associated with the prolonged response period (median DOR of higher CD62L^+^ CD27^+^ CD8^+^ CAR-T cells vs. lower: 27.2 vs. 9.4 months) (**Figure 2**).

#### 3.5.4 Allogeneic BCMA-Targeted CAR-T-Cell Products

Patients with relapsed or refractory hematological malignancies who are suitable to be treated by CAR-T-cell therapy usually have a high tumor load and many deficiencies in their T-cell population (76). These limitations increase the difficulty of obtaining sufficient qualified T cells as the materials for CAR-T cells’ manufacture after apheresis (76). Up to now, allogeneic CAR-T-cell therapy has shown a certain extent efficacy for the patients who have appropriate donors (77, 78). However, allogeneic materials bring the risk of graft-versus-host disease (GvHD) undoubtedly (79). The most effective measure at present is using gene-editing technology such as zinc-finger nucleases (ZFN), transcription activator-like effector nuclease (TALEN) technology, and the CRISPR/Cas9 system to knockout T-cell receptors (TCR) at the DNA level to reduce the risk of GvHD (80). Moreover, many novel strategies such as RNA silencing (80) and membrane protein intracellular retention technology have been used to knockdown TCR at mRNA or other levels. ASH2021 updated two novel allogeneic BCMA-targeted CAR-T-cell products: ALLO-715 (81) and CYAD-211 (82). ALLO-715 used Collectis TALEN technology to disrupt the TCR alpha constant (TRAC) and CD52 gene, which required multiple operating steps and longer culture time to increase the exhaustion of T cells. By comparison, CYAD-211 converted to use short hairpin RNA (shRNA) to knockdown TCR expression at mRNA level, which could reduce the preparation duration. The shRNA in CYAD-211 was coexpressed with its CAR, so it required only one step to achieve the genetic modification. The phase I trials of these two, named UNIVERSAL and IMMUNICY-1 respectively, have already begun, and the former provided more details to us. A report in ASH2020 about UNIVERSAL suggested that a higher dose level of ALLO-715 could improve clinical efficacy. After the 320/480 × 10^6^ CAR-T-cell infusion and 7.4 months median follow-up, the ORR (*n* = 26) was 61.5%, and the rate of VGPR was 38.5%. The incidence rate of CRS was 52.4%, and just one of them was rated level 3. Among ten patients who have been tested, eight obtained negative results of MRD, proving the efficacy of ALLO-715 to some extent. Regrettably, information about GvHD did not present here (81). As for IMMUNICY-1, none of the nine enrolled participants showed GvHD after CYAD-211 inputting. However, the grafts only lasted 3 to 4 weeks *in vivo*, which can be interpreted as the rejection of patients’ healing immune system. Moreover, the effectiveness of CYAD-211 needs further assessment. There is also a clinical study showing that allogeneic CAR-T cells from the same donor were used as one of the preprocessed methods for subsequent allogeneic hematopoietic stem cell transplantation (Allo-HSCT) (83). The results showed that this strategy was effective in treating patients with MM who had relapsed after multiple LOTs. The conclusions of these studies, along with part of previous findings (84–87), demonstrated the prospect of allogeneic BCMA-targeted CAR-T-cell therapy and its clinical availability as an adjuvant treatment in combination with other traditional or neoteric therapeutic methods such as allo-HSCT. Rather than stop here, we need more in-depth studies in this area. We think allogeneic CAR-T-cell therapy can be seen as a “stepping stone”, and the development and improvement of universal CAR-T cell will become mainstream one day.

#### 3.5.5 Bispecific BCMA-Targeted CAR-T Cell

Although BCMA-targeted CAR-T-cell therapy has shown a favorable efficacy, the disease recurrence after this agents’ treatment remains a critical concern (88). One of the reasons of palindromia is the tumor cells’ immune escape, which is induced by the adaptive decrease of BCMA expression after long-term treatment, the amplification of a small number of BCMA-negative minimal residual lesions surviving from the lethal effect of CAR-T cells, or other mechanisms (89, 90). To solve this question, an increasing number of studies on dual-targeting or combined-targeting CAR-T cells have been carried out (91, 92). ASH2021 updated two novel bispecific BCMA-targeted CAR-T-cell products: the first targets two tumor-associated antigens (TAAs) (BCMA and CD24) (93), and another one constructs a synthetical CAR targeting the pan-TAAs, containing MHC class I polypeptide‐related sequence A/B (MICA/MICB), as the companion target of the classic target BCMA (94). Previous studies showed us that tumor-initiating cells’ (TICs) survival and amplification after CAR-T-cell therapy could seed relapse by acquiring the resistance. Part of these cells were the CD24^+^BCMA^−^ subgroups (95). As expected, these BCMA-CD24-targeted CAR-T cells, which target and kill TICs effectively, can be activated by exposing to the CD24^+^ microenvironment. When CD24^+^ MM cells (ARP-1 CD24OE or OCI CD24OE cells) were co-cultured with these CAR-T cells *in vitro* with the 5:1 proportion of the CAR-T cells and the MM cells, the clearance rates of ARP-1 CD24OE and OCI CD24OE cell lines were 99% and 89%, respectively. Unlike the CAR mentioned above targeting two well-defined epitopes, another bispecific CAR targets BCMA and a pan-TAAS simultaneously. The conserved α3 domain of MICA/MICB is the target of the CAR, which could drive antitumor immunoreaction and prevent MICA/MICB shedding at the same time (96). These studies elucidated that both BCMA and the additional antigens could activate those bispecific CAR-T cells targeting them, making these artificial immune cells degranulated to exert their cytotoxicity. Bispecific BCMA-targeted CAR-T-cell therapy is a promising strategy to expand the splash radius of CAR-T cells, which is expected to reduce the resurgence of MM after CAR-T-cell therapy.

#### 3.5.6 The Novel Ameliorative Methods for BCMA-Targeted CAR-T Cell

**Figure 3:**
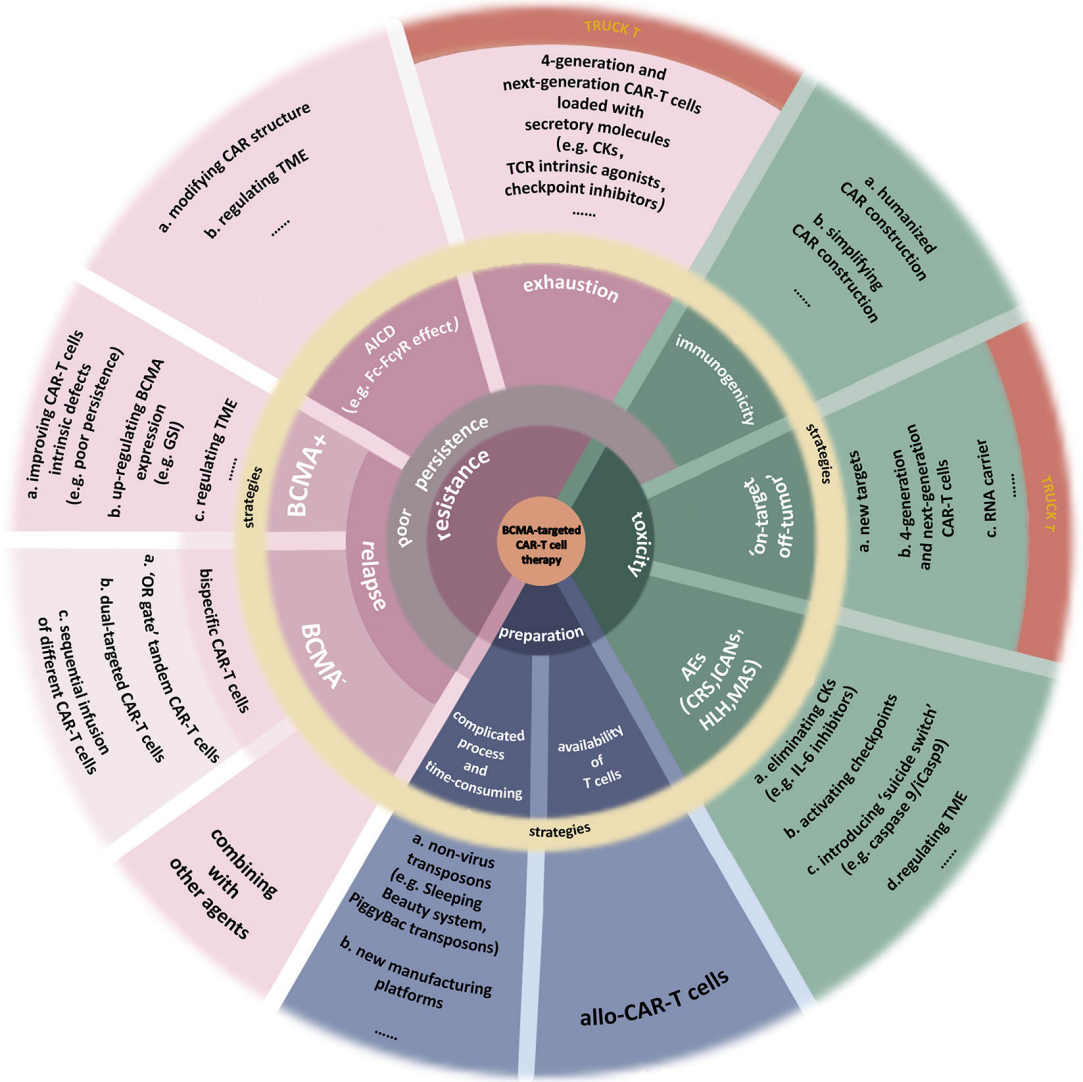
Limitations of BCMA-targeted CAR-T-cell therapy. This image summarizes the deficiencies of BCMA-targeted CAR-T-cell therapy. Also, part of improvements to address these limitations is presented. **(a)** Toxicities. Nonhuman single-chain variable fragments (scFv) in classical CAR construction increases the heterogeneity of CAR-T cell, inducing attack by the immune system of patients. Using humanized materials to prepare CAR or simplifying the CAR construction can reduce the heterogeneity. Finding new targets with higher specificity can reduce the “on-target off-tumor” effect. Fourth-generation and next-generation CAR-T (TRUCK T) cells, fitted with transgenic “payloads” which can express specific secretory molecules or membrane receptors, create a more favorable microenvironment for their function. BiTE-armored and chemokine receptor-armored CAR-T cells can target tumor cells more precisely. The adverse events (AEs) after CAR-T-cell therapy include cytokine release syndrome (CRS), immune effector cell-associated neurotoxicity syndrome (ICANs), hemophagocytic lymphohistiocytosis (HLH), macrophage activation syndrome (MAS), or more. The most common of them is CRS. Clearance of excess cytokines (CKs) is the key to addressing these toxicities. **(b)** Resistance. Resistance to CAR-T-cell therapy induces the disease recurrence, including BCMA^+^ and BCMA^−^ relapse. Multiple factors, both internal and external of the tumor, may cause malignant downregulation of BCMA, making it insufficient to be recognized by CAR-T cells. Bispecific CAR-T-cell therapies, including “OR gate” tandem CAR-T cells, dual-targeted CAR-T cells, and sequential regimens, have been used to address BCMA^−^ relapse and the off-target effect. As for the BCMA^+^ relapse, it can be caused by multiple factors from CAR-T cells, myeloma cells, even TME. A severe problem of existing CAR-T cells is their poor persistence. There are many reasons for this issue, such as the CAR-T-cells’ exhaustion or the clearance to these artificial immune cells, which are similar to physiological activation-induced cell death (AICD). The hinge domain of CAR has a similar structure to the Fc domain in Ig. This characteristic induces the Fc-FcγR interactions between CAR-T cells and other immune cells, killing CAR-T cells. Studies have done to improve CAR’s structure by modifying the spacer, such as extending the hinge domain or finding a novel hinge with a lower affinity for FcγR so that to avoid immune system cleanup to activated CAR-T cells. In addition, some inhibitors in TME, the expression of specific inhibitory genes, or the increase of terminal CD45RA^+^ cells all cause the AICD-like effects, thus reducing the persistence of CAR-T cells. T cells stemness is closely associated with the efficacy and exhaustion of CAR-T cells. TRUCK T cells produce specific secretory molecules such as some CKs, which could increase the stemness of cells (e.g., IL-15), TCR intrinsic agonists (e.g., 4-1BB), or checkpoint inhibitors (e.g., PD-1 inhibitors). As a result, the persistence of these next-generation CAR-T cells has been dramatically improved. **(c)** Preparation process. The complicated preparation process of CAR-T cells is time-consuming, and the quality of T cells as the materials sometimes is not up to par. Developing new nonlentivirus transposons such as PiggyBac transposons or culturing platforms can improve manufacturing efficiency. Using allogeneic T cells as the materials can improve the quality of T cells but induce graft-versus-host disease (GvHD).

BCMA-targeted CAR-T-cell therapy also has some other deficiencies (6, 97), which call for reasonable solutions (**Figure 3**). ASH2021 updated several novel engineering improvements to address part of these limitations.

##### 3.5.6.1 CAR-T Cells’ Poor Persistence

CAR-T cells’ poor endurance is a major cause of disease recurrence after treatment (98). By testing peripheral blood and bone marrow samples from the patients treated with BCMA-targeted CAR-T cells, a study in ASH2021 (99) found that increased BCL-XL expression may enhance CAR-T cells’ resistance to the similar effect like activation induced cells death (AICD), and prolong these cells’ persistence through responding to CD28 costimulatory signals. Based on this discovery, the researchers designed a second-generation lentiviral CAR (BCMA-BCL2L1-CAR)-armored BCL-XL. In the edited gene of this CAR, classical anti-BCMA scFV-41BBz CAR and BCL2L1 cDNA were linked by a self-cleaving 2A sequence. This kind of modified BCMA-targeted CAR-T cell has a higher BCL2L1 expression, and in MM cell lines (MM1S, OCMY5, and H929) expressing the ligands of FAS death receptor (FASLG), BCMA-BCL2L1-CAR-T cells observably outperformed unarmored BCMA-CAR-T cells in terms of viability and cytolysis activity. Moreover, BCMA-BCL2L1-CAR-T cells with less cells exhaustion showed greater ability to kill the tumor cells under chronic antigenic stimulation, which could cause AICD more easily.

For extending the duration of BCMA-targeted CAR-T cells, a nonvirus transposon system called PiggyBac (PB) has already been put into use. Two novel CAR-T-cell products targeting BCMA manufactured by PB were reported in this meeting. They were named P-BCMA-101 (autologous) and P-BCMA-ALLO1 (allogeneic), respectively (100). This study proved that PB does not only sped up the preparing process of CAR-T cells but also could preserve more desirable stem cell memory T cells (Tscm), whose proportion were closely related to the persistence of BCMA-targeted CAR-T cells (101, 102). Results of the phase I/2 clinical trial for P-BCMA-101 named PRIME (NCT03288493) certified the safety of this agent.

##### 3.5.6.2 CAR-T Cells’ Immunogenicity

The nonhuman sequences in scFvs of anti-BCMA CAR have immunogenicity, which can trigger the host versus graft (HvG) response (76). A study reported in ASH2021 constructed a new anti-BCMA CAR (FHVH33-CD8BBZ) that replaced the normal scFv with a smaller fully human BCMA-targeted heavy-chain variable domain (FHVH33) (103). Because of the lack of light chain, artificial linker, and two linker-associated junctions in scFv, FHVH33 may have lower immunogenicity. After infusing this novel CAR-T cell (FHVH33-T), the ORR was 92% (23/25) and 68% of all patients (17/25) got better than VGPR. Up to the date, the DOR was 50 weeks at the highest two-dose levels (4/6 × 10^6^ CAR^+^ T cells/kg), and the overall median PFS was 78 weeks. Assessing the blood CAR^+^ cells confirmed that the median peak blood CAR^+^ cell level was 126.5 cells/µl, and the median time postinfusion of peak blood CAR^+^ cell levels was 10.5 days. These results suggest that the immunogenicity of CAR-T cells can be reduced to some extent by fully humanizing and reducing the molecular size of CAR, thereby reducing the likelihood of HvG effect and prolonging CAR-T-cell retention *in vivo*.

##### 3.5.6.3 Availability of Autologous T Cells

Various anterior treatments to the patients with RRMM disable T cells and develop adverse phenotypes, such as exhaustion and senescence (104, 105). These, together with the immunosuppressive characteristic of TME (98), reduce the availability of the heavily pretreated patients’ T cells to be the materials for CAR-T cells’ preparation. To solve this question, a study explored whether T cell materials with better quality derived from a similar preconditioning approach of autologous hematopoietic stem cell transplantation (HSCT) could be used to prepare CAR-T cells (106). The basic process is collecting CD34^+^ progenitor cells from peripheral blood of patients and mobilizing them by the granulocyte-colony stimulating factor (G-CSF) in earlier stages of MM treatment, then reserving them for the preparation of BCMA-targeted CAR-T cells. The results showed that pretreatment by G-CSF did not have significant negative effects on T cells. It is a pity that there is no mention in this report of CAR-T cells being produced in this way.

#### 3.5.7 Effects of Other Factors on BCMA-Targeted CAR-T-Cell Therapy

##### 3.5.7.1 Corticosteroids

The side-effects of CAR-T cell therapy, such as CRS, ICANS, macrophage activation syndrome (MAS), and hemophagocytic lymphohistiocytosis (HLH), need to be controlled by tocilizumab, corticosteroids, and, or anakinra (107–109). However, steroids not only suppress the excessive inflammatory response but also inhibit T cells’ activity and may reduce the efficacy of CAR-T-cell therapy (110). Previous studies have analyzed the effects of steroids on CAR-T-cell therapy in some other hematologic malignancies with impure results (80). A study (111) in ASH2021 compared the therapeutic effects of BCMA-targeted CAR-T cells combined with or without steroids in patients with RRMM. After using steroids 4 days medially, the results showed that there were no significant differences in ORR (95.8% vs. 84.2%), PFS (13.1 vs. 13.2 months), OS (not reached vs. 26.4 months), and time-to-next treatment (TTNT) (10.5 vs. 7.0) between the “experimental group” that received steroids and the “control group.” Moreover, these indicators were not affected by steroids obviously at different doses (0, ≤60, and >60 mg). It is worth mentioning that more than 5 days use of steroids may affect PFS and TTNT to some extent (TTNT/PFS after 0, 1–5, and ≥5 days steroids: 22.8, 24.6, and 12.5 months/13.2, 21.4, and 10.6 months). Although this study seems to prove that cortisol use does not affect CAR-T- cell therapy in general, it did not follow the principles of controlled trials strictly, so the conclusions are open for debate.

##### 3.5.7.2 NKTR-255

In addition to radically improving the structure of CAR-T cells, many other attempts have been made to address their poor persistence (112). NKTR-255, a recombinant human IL-15 (rhIL-15) receptor agonist, can activate the IL-15 pathway and promote the proliferation of memory CD8^+^ T cells and Tscm subsets in tumor-specific T-cell colonies (101, 102). Recently, a phase I study about the influence of NKTR-255 is ongoing, and ASH2021 provided us with the preliminary results (113). The T/CAR-T cell counts and Ki67 expression of six enrolled patients treated with CAR-T/CAR-NK before were evaluated to assess T cells’ viability before and after the NKTR-255 administration. After treating by NKTR-255, the peak number of CD3^+^ CAR-T cells in peripheral blood of three patients increased by 70% compared with the baseline, and the ratio of CD4^+^:CD8^+^ CAR-T cells had changed in one patient with a ~2-fold increase in CD8^+^ compared with CD4^+^ CAR-T cells. Following one dose of NKTR-255, all patients had an average of ~1.6-fold increase in total CD8^+^ T cells and an average 9-fold increase in the percentage of Ki67^+^CD8^+^ T cells, standing up for the role of NKTR-255 in saving the prostrated CAR-T cells. This study demonstrated the feasibility of combining drugs to prolong CAR-T-cell persistence.

##### 3.5.7.3 Gamma Secretase Inhibitor

As mentioned above, the formation of sBCMA through gamma secretase reduces the expression of BCMA on MM cells, making them escape from BCMA-targeted CAR-T cells’ lethal effect (114, 115). Those sBCMA in circulation may also interfere the therapeutic process of CAR-T cells for patients with RRMM (116). Gamma secretase inhibitors (GSI) can increase BCMA density on the surface of tumor cells and decrease the level of sBCMA, reinforcing the efficacy of the therapies targeting BCMA in murine models with MM (117). Based on these findings, a phase I human trial of GSI (JSMD194) in combination with BCMA-targeted CAR-T-cell therapy has done and was reported in ASH2021 (118). This trial enrolled 18 patients who had received a median of 10 prior LOTs. After three oral doses (25 mg) administered 48 h apart over 5 days of JSMD194 monotherapy, the median number of the receptors on each tumor cell increased from 610 to 9,563, which was 12 times as large as before. These patients were treated with different-dose BCMA-targeted CAR-T cells subsequently. The resulting ORR was 89%, with 44% of all patients achieving CR (including 27% with sCR) and 77% getting better than VGPR. The median PFS reached 11 months with a median of 20 months follow-up. These data illustrated that the combination of GSI and BCMA-targeted CAR-T-cell therapy were safe and tolerable with an improved antitumor effect, even at very low doses of CAR-T cells.

##### 3.5.7.4 Extrinsic and Intrinsic Factors of Tumor

Using BCMA-targeted CAR-T cells to treat RRMM patients with huge differences between individuals can lead the divergent outcomes. Both intrinsic and extrinsic factors of the tumor, such as the expression of tumor genomics or the immunosuppressive elements presented in TME (119, 120), may contribute to the vast gap between these therapeutic results (88). There were two studies in ASH2021 exploring the relationship between these factors and the therapeutic effects of BCMA-targeted CAR-T cell therapy. The first study (121) used mass cytometry (CyTOF) to longitudinally analyze the immunophenotype of peripheral blood mononuclear cells (PBMC, CD45^+^CD66b^−^) from the patients treated with ide-cel and found that the phenotypic changes of PBMCs along with the CAR-T cells’ expansion: CD14^+^ monocytes declined (40% to 13%) while CAR^−^CD8^+^ T cells, which differentiated towards a CD8^+^ effector-memory phenotype (EM, CCR7^−^CD45RA^−^), expanded (32% to 43%) from weeks 0 to 4 after the infusion of CAR-T cells. However, the BM samples from the patients who relapsed after CAR-T-cell therapy showed a reversal trend: CD14^+^ monocytes remained invariable or slightly elevated, but CAR^-^CD8^+^ T cells decreased instead. This study also analyzed the BM mononuclear cells (BMMC) from patients with ide-cel therapy by unbiased mRNA profiling using single-cell RNA-seq (scRNA-seq). The outcome revealed that patients who relapsed had an altered gene expression, suggesting that the intrinsic tumor factors had an impact on CAR-T-cell therapy. For example, upregulation of gene expression like proinflammatory chemokines (CCL3, CCL4), antiapoptotic genes (MCL-1, FOSB, JUND), and NF-kB signaling genes (NFKBIA) could promote relapse, which may be one of the mechanisms for the resistance to CAR-T therapy. Interestingly, another study (122), which also used scRNA-seq to compare the BM and PBMC samples from the patients who relapsed within 1 year [early relapse (PD)] or more than 1 year [durable response (DR)] after BCMA-targeted CAR-T cells’ infusion, showed that the DR patients had more BCMA-high CD138^+^ cells compared with the PD patients. Moreover, there were two unique clusters in DR patients’ CD138^+^ cells while only one in PD patients. The top marker genes in these three clusters were associated with the pathway of IL-15 signal, BCR signal, and the primary immunodeficiency signal. It should be added that the patients achieving more than VGPR after CAR-T-cell therapy had a higher proportion of CD8^+^ T cells compared with poor responders (<VGPR) (37% vs. 11%), a lower proportion of CD14^+^ monocytes (30% vs. 61%) and NK cells (2% vs. 6%) in PB (121).

##### 3.5.7.5 CAR Density

Up to now, the underlying mechanisms of CAR-T cells’ dysfunction are not well understood. A part of the studies has proved that the density of CAR can affect the availability and antitumor effectiveness of CAR-T cells. A recent study (123) in ASH2021 performed genomic and functional analyses on the BCMA-targeted second-generation CAR-T cells which have 4-1BB costimulatory domains with different CAR densities (CAR_High_ and CAR_Low_). The genomic analysis showed entirely different profiles between CAR_High_-T cells and CAR_Low_-T cells in both CD4^+^ and CD8^+^T-cell subsets, with 3,500-fold difference in gene expression. These genes were related to T-cell activation, and the tonic signaling in CAR_High_-T cells associated with T-cell proliferation or exhaustion. The functional analysis showed that before encountering the antigens, CAR_High_-T cells presented intensive tonic signaling, which led to higher activation and more differentiation. After identifying their targets, CAR_High_-T cells released an increased number of cytokines, indicating that they would exert more potent cytotoxic effects. Moreover, in these CAR_High_-T cells, the factors about cell proliferation and exhaustion (PD1^+^/LAG3^+^/TIGIT^+^) were increased as well, and these cells presented a higher percentage of terminally differentiated T cells (CCR7^-^/CD45RA^+^). The regulons associated with NR4A1 transcription factor that promotes T-cell exhaustion (124) also have been activated in CAR_High_-T cells. By contrast, the analysis of CAR_Low_-T cells demonstrated that they had better persistence, in which more CCR7^+^/CD45RA^+^/CXCR3^+^ Tscm were retained. That is to say, increasing CAR density could enhance CAR-T cells’ activation, differentiation, and cytotoxicity but reduce their long-term efficacy. Therefore, the CAR density may play a crucial role in CAR-T cells’ persistence. It is expected to promote the effectiveness of CAR-T-cell therapy by rationally using the engineered T cells with different CAR densities.

### 3.6 Prospects for CAR-NK Cell Therapy in MM

Since NK cell activation does not need the prior antigen stimulation and strict HLA matching, CAR-NK cell therapy has shown its unique competitiveness under this era of rapid development of cellular immunotherapy (125). Compared with CAR-T-cell therapy, it seems to have better safety. Because NK cell cytotoxicity is mediated by releasing perforin and granulocytase rather than cytokines such as IL-1, IL-2, IL-6, TNF-α, IL-8, IL-10, and IL-15 released by CAR-T cells, or expressing the apoptosis-inducing ligands including Fas Ligand (FasL) and (TNF)-related apoptosis-inducing ligand (TRAIL), they rarely cause CRS and neurotoxicity (125). In addition, CAR-NK cells are more suitable to be the “off-the-shelf” therapy than CAR-T cells. According to the available studies, allogeneic CAR-NK cells almost never induce GvHD, and the source of NK cells is more extensive, they can be differentiated from peripheral blood (PB) cells, umbilical cord blood (UCB) cells, embryonic stem cells (ESCs), induced pluripotent stem cells (iPSCs), and specific NK cell lines such as NK92 cells (126). For MM, a variety of CAR-NK cells have been studied in preclinical or clinical trials. They target different targets, including BCMA, CD138, and CS1 (CD319/SLAMF7) (125). Up to now, clinical trials have been conducted on two anti-BCMA CAR-NK cells derived from umbilical/cord blood (CB) (NCT05008536) and NK92 cell line (NCT03940833), but they have not published the relevant data yet. Current studies are focused on optimizing existing BCMA-targeted CAR-NK cells and developing universal CAR-NK cell therapy derived from iPSCs. Existing CAR-NK cell products have been genetically modified by gene editing (127), mRNA electroporation (128) and other techniques, which significantly increased their targeting specificity and tumor killing effectiveness. A study in ASH2021 (129) creatively combined three antitumor modalities, including CAR, TCR, and CD16 Fc receptor, which is naturally expressed on NK cells. By engineering them into iPSC-derived T cells, they demonstrated the synergistic effect of this tri-modal CAR-iT cell in overcoming tumor cell escape and their heterogeneity. In the future, if we want to promote the clinical application of CAR-NK cells, it is necessary to properly solve or evade their existing limitations such as short life, low toxicity, and the off-target effect.

## 4 Discussion

Although multiple kinds of BCMA-targeted immunotherapies, including ADCs, BsAbs, and adoptive cell therapies have presented gratifying results of their primeval clinical trials, there are still many hurdles that need to be overcome before they go into real-world service to benefit more suffering patients with RRMM. According to the reports in ASH2021, BCMA-targeted CAR-T-cell therapy seems to show better efficacy than other agents. However, we cannot simply judge the merits of these products. The unique characteristics of these agents not only grant them irreplaceable advantages but also give them inevitable limitations. For instance, anti-BCMA CAR-T-cell therapy with better performance requires more complex preparation conditions and more expensive treatment costs, which are difficult for ordinary families to afford (130). For BsAbs, because of the relatively short half-life period (131), they need to extend the infusion time or improve the medication frequency to maintain its efficacy (132), which also increases the costs of treatment. BiTE depends on the quality of T cells, thus it is mainly used for front-line treatment (133, 134). As for those off-the-shelf ADCs which are cheaper and more convenient, they also have to be administered more frequently because they take effect by internalizing them into the tumor cells and releasing payloads, which are easy to be cleared by the intracellular active substances (135). Luckily, for these agents, the ameliorations for deficiencies are thought in more detail as well. Take the CAR-T products for example, concerning the long-term consumption in preparation of the costly CAR-T cells, multiple new techniques such as Piggy Bac and Sleeping Beauty transposition system have been put into study. Of course, these deficiencies are just a drop in the bucket. Therefore, with continuing the existing studies, we also need to study the underlying mechanisms that influence the curative effect to optimize BCMA-targeted immunotherapies. We have to say that the development of various immunotherapy methods in recent years has changed the treatment landscape of MM to some extent. In the face of so many biological drugs, formulating appropriate medication regimens will be a challenge for clinicians (136). Existing anti-BCMA agents are primarily used to treat those adults with RRMM who have received more than 4 LOTs, but the studies about their front-line application are limited. In fact, the patients who have received three or more LOTs have worse physical conditions, therefore, moving the treatment window forward moderately may be the direction of future clinical studies. Although the prognosis of MM patients has improved greatly, the refractory phenotypes such as EMD are still difficult to overcome. To solve these problems, laboratory research and enriching our clinical experience should continue simultaneously. We believe that with the further research, RRMM patients will eventually go through their winter.

## Author Contributions

RG is a major contributor in writing the manuscript. WL has made substantial contributions to the conception. RG and XJ drafted the work. YZ, XC, and XJ have substantively revised it. MZ reviewed the draft. All authors read and approved the final manuscript.

## Funding

This work was supported by grants from the General Project of National Natural Science Foundation of China (81970180 to MZ) and the Key Science and Technology Support Project of Tianjin Science and Technology Bureau (20YFZCSY00800 to MZ), as well as Tianjin Key Medical Discipline (Specialty) Construction Project.

## Conflict of Interest

The authors declare that the research was conducted in the absence of any commercial or financial relationships that could be construed as a potential conflict of interest.

## Publisher’s Note

All claims expressed in this article are solely those of the authors and do not necessarily represent those of their affiliated organizations, or those of the publisher, the editors and the reviewers. Any product that may be evaluated in this article, or claim that may be made by its manufacturer, is not guaranteed or endorsed by the publisher.

## Glossary

MM: multiple myeloma
PI: proteasome inhibitor
IMiD: immunomodulatory drug
mAbs: monoclonal antibodies
BCMA: B-cell maturation antigen
ADCs: antibody-drug conjugates
BITEs: bispecific T-cell engagers
CAR-T cell: chimeric antigen receptor-T cell
ASH: American Society of Hematology
PCs: plasma cells
APRIL: a proliferation-inducing ligand
NF-κB: nuclear factor kappa-B
RAS/MAPK: rat sarcoma/mitogen-activated protein kinase
PI3K-PKB: phosphoinositide-3-kinase–protein kinase B
JNK: c-Jun N-terminal kinase
TEM: tumor microenvironment: H3K36me3
H3K36 trimethylation: sBCMA
soluble BCMA: SLAMF7
signaling lymphocytic activation molecule family member 7: LOT
prior lines of therapy: RRMM: relapsed or refractory multiple myeloma
PD: pharmacodynamics
IRR: infusion-related reaction
DLT: dose-limiting toxicity
VGPR: very good partial responses
ORR: objective response rate
AEs: adverse events
DEX: dexamethasone
PK: pharmacokinetics
BM: bone marrow
BsAbs: bispecific antibodies
BiTEs: bispecific T-cell engagers
TCR: T-cell receptors
GPRC5D: G-protein coupled receptor C family 5D
pts: patients
LEN: lenalidomide
POM: pomalidomide
NA: not applicable
DOR: duration of response
TRAEs: treatment-related AEs
CRS: cytokine release syndrome
Gr: grade
ICANS: immune effector cell-associated neurotoxicity syndrome
TEAEs: treatment-emergent AEs
NK: natural killer
TriTAC: tri-specific T-cell activation constructs
MTD: maximum tolerated dose
RP2D: recommended phase two dose
MMAF: monomethyl auristatin-F
FDA: Food and Drug Administration
MoABs: anti-CD38 antibody
BCVA: best-corrected visual acuity
OS: overall survival
sCR: stringent complete response
cilta-cel: ciltacabtagene autoleucel
MRD: minimal residual disease
RWCP: real-world clinical practice
CR: complete response
ide-cel: idecabtagene vicleucel
HRQoL: health-related quality of life
sAMT: subsequent antimyeloma therapy
scFvs: single-chain variable fragments
GvHD: graft-versus-host disease
TRAC: TCR alpha constant
shRNA: short hairpin RNA
Allo-HSCT: allogeneic hematopoietic stem cell transplantation
TAA: tumor-associated antigens
MGUS: monoclonal gammopathy of undetermined significance
TICs: tumor-initiating cells
AICD: activation-induced cells death
FASLG: FAS death receptor ligand
G-CSF: granulocyte-colony-stimulating factor
PB: PiggyBac
MAS: macrophage activation syndrome
HLH: hemophagocytic lymphohistiocytosis
rhIL-15: recombinant human IL-15
GSI: gamma secretase inhibitors
PBMA: peripheral blood mononuclear cells
BMMC: BM mononuclear cells
scRNA-seq: single-cell RNA-seq
CKs: cytokines
AICD: activation-induced cell death
PFS: progression-free survival
HvG: host versus graft
TALEN: transcription activator-like effector nuclease
MICA/MICB: MHC class I polypeptide‐related sequence A/B
FasL: Fas ligand
TRAIL: (TNF)-related apoptosis-inducing ligand
PB: peripheral blood
UCB: umbilical cord blood
ESCs: embryonic stem cells
iPSCs: induced pluripotent stem cells
CB: cord blood
EMD: extramedullary disease
